# Reviewing the Evidence Base for Topical Steroid Withdrawal Syndrome in the Research Literature and Social Media Platforms: An Evidence Gap Map

**DOI:** 10.2196/57687

**Published:** 2024-12-06

**Authors:** Noreen Orr, Morwenna Rogers, Abigail Stein, Jo Thompson Coon, Kenneth Stein

**Affiliations:** 1 University of Exeter Medical School Exeter United Kingdom

**Keywords:** topical steroid withdrawal syndrome, evidence gap map, social media, blogs, Instagram, Reddit, topical corticosteroids

## Abstract

**Background:**

Within the dermatological community, topical steroid withdrawal syndrome (TSWS) is a medically contested condition with a limited research base. Published studies on TSWS indicate that it is a distinct adverse effect of prolonged use of topical corticosteroids, but there is a paucity of high-quality research evidence. Among the “patient community,” awareness has been increasing, with rapid growth in social media posts on TSWS and the introduction of online communities such as the International Topical Steroid Awareness Network. This evidence gap map (EGM) was developed in response to recent calls for research to better understand TSWS and aims to be an important resource to guide both researchers and clinicians in the prioritization of research topics for further research.

**Objective:**

This study aims to identify the range, extent, and type of evidence on TSWS in the research literature and social media platforms using an EGM.

**Methods:**

The MEDLINE and Embase (Ovid), CINAHL (EBSCOhost), and ProQuest Dissertations & Theses and Conference Proceedings Citation Index (CPCI-Science and CPCI-Social Science & Humanities via Web of Science) databases were searched. The final search was run in November 2023. Study titles, abstracts, and full texts were screened by 2 reviewers, and a third was consulted to resolve any differences. Blogging sites WordPress, Medium, and Blogspot and Google were searched; Instagram and Reddit were searched for the 100 most recent posts on specific dates in February 2023. Blog titles, Instagram posts, and Reddit posts were screened for relevance by 2 reviewers. A data extraction tool was developed on EPPI-Reviewer, and data extraction was undertaken by one reviewer and checked by a second; any inconsistencies were resolved through discussion. We did not undertake quality appraisal of the included studies. EPPI-Reviewer and EPPI-Mapper were used to generate the interactive EGM.

**Results:**

Overall, 81 academic publications and 223 social media posts were included in the EGM. The research evidence mainly addressed the physical symptoms of TSWS (skin), treatments, and, to a lesser extent, risk factors and disease mechanisms. The social media evidence primarily focused on the physical symptoms (skin and nonskin), mental health symptoms, relationships, activities of everyday living, beliefs and attitudes, and treatments.

**Conclusions:**

The EGM shows that research evidence is growing on TSWS but remains lacking in several important areas: longer-term prospective observational studies to assess the safety of prolonged use of topical corticosteroids and to prevent addiction; qualitative research to understand the lived experience of TSWS; and longitudinal research on the patient’s “TSWS journey” to healing. The inclusion of social media evidence is a methodological innovation in EGMs, recognizing the increased presence of #topicalsteroidwithdrawal on social media and how it can be used to better understand the patient perspective and ultimately, provide better care for people with TSWS.

## Introduction

### Background

Topical corticosteroids (TCs) are prescribed for many skin disorders, including chronic conditions such as atopic dermatitis, the most common form of eczema. That they have been the mainstay of treating atopic dermatitis for >60 years is reflected in the many international guidelines published for managing atopic dermatitis [1]. TCs are included in these guidelines as one of the several treatment options, with recommendations provided for dosing, frequency of application, maintenance, and screening for side effects. In a comparison of 14 international guidelines from across the globe published between 2007 and 2018, LePoidevin et al [1] found that the fingertip unit (approximately 0.5 g) as the method to guide TC application was recommended in over half. Most guidelines recommended once to twice daily application to control flares, and most recommended using TCs as maintenance therapy to reduce the incidence of flares. Just over half recommended screening for cutaneous side effects, and none of them recommended screening for systemic side effects of TCs. Despite the variation across the guidelines, importantly, all acknowledged that the prescription of TCs should be tailored to the unique characteristics of individual patients [1].

While TCs have been effective for many people [2-4], they have also been associated with the risk of adverse effects, particularly when used long term [3]. Cutaneous side effects include atrophy, striae, rosacea, perioral dermatitis, acne, and purpura [2,4-7], and systemic side effects include adrenal insufficiency, Cushing syndrome, glaucoma, and cataract [2,3,6,7]. In the recently updated guidelines from the American Academy of Dermatology for the management of adult atopic dermatitis with topical therapies, the “related concepts” of topical steroid addiction (TSA) and topical steroid withdrawal (TSW) are mentioned, along with red face syndrome and red scrotum syndrome, as adverse side effects that may occur after prolonged use of TCs [4]. The European Guideline (EuroGuiDerm) on atopic eczema also has 1 mention of “corticosteroid addiction syndrome” as an important side effect of TCs [7].

The concepts of TSA and TSW are often used interchangeably in the literature [8,9], but they coalesce around 2 elements: physical dependence on TCs and worsening skin symptoms after withdrawal [9-11]. Studies have shown that people use an increased quantity and potency of TCs for months to years to control their skin condition (it is not clear whether the decision to increase the quantity and potency of TCs was made by the individuals themselves or following advice from a health professional) [11,12]. Then, on withdrawal from TCs, individuals experience the rebound phenomenon, often with more extensive and more severe skin manifestations than with the original skin condition [9,13]. An example of a withdrawal symptom is red skin syndrome, characterized by severe erythema in various areas of the body, along with burning and stinging [11]. Withdrawal from TCs can mean complete cessation or tapering use by reducing potency and applying less often and to fewer parts of the body. Following the International Topical Steroid Awareness Network, we use the term topical steroid withdrawal syndrome (TSWS), which is an “umbrella term” for the condition, as it encompasses symptoms before (TSA) and after cessation of TCs (red skin syndrome and TSW).

Despite the recognition of the “addiction” effects of TCs in 1969 [14], TSWS is controversial today [9,15], as “doubts still exist whether this condition is legitimate” [16]. There are no clear or accepted diagnostic criteria for TSWS and for many clinicians, it can be challenging to distinguish it from other conditions (eg, allergic contact dermatitis) [9,17], and some see it as an “exacerbation of the underlying skin disease” [18]. That said, in 2021, the National Eczema Society and the British Association of Dermatologists [19] published a joint statement on TSWS, and in the same year, the Medicines and Healthcare products Regulatory Agency (MHRA) issued a drug safety update advising patients to seek medical help if experiencing redness, burning, itching, or stinging of the skin on stopping TCs [20]. The MHRA requested that warnings and precautions for use on TCs be added to the summary of product characteristics and patient information leaflet for prescription-only and pharmacy-only medicines. The National Eczema Association in the United States has also recognized TSWS as a potentially serious side effect of using TCs [21], as has DermNet in New Zealand [22].

Although the MHRA described TSWS as rare, it is estimated to occur in approximately 12% of people with atopic dermatitis who use TCs [13]. Evidence for the increasing awareness of TSWS is demonstrated by the rapid growth in social media posts and discussions on TSWS [23-25] and the introduction of online communities, such as the International Topical Steroid Awareness Network and Scratch That, United Kingdom. It suggests that people living with TSWS are using social media for information and support, particularly as patients have reported feeling ignored, dismissed, or blamed by their physicians for inappropriate use of TCs [26,27]. However, for many dermatologists and clinicians, social media is perceived negatively as a key source of misinformation [15,28] and a driver of patient self-diagnosis [29]. In contrast, Bowe et al [24] suggested that an awareness of the influence of social media platforms on perceptions of TCs could help “bridge the doctor-patient gap”, and arguably, it offers one way to gain a greater understanding of people’s experiences of living with the symptoms of TSWS: “[p]atients want professionals to recognise the impact of symptoms experienced and feel that recognition would be an important first step to better management” [27].

### Aim

This study aimed to identify the range, extent, and type of evidence on TSWS in the research literature and compare the topics identified with the subjects of posts on a number of social media platforms. The overall objectives of the study were as follows:

- Identify published evidence on TSWS: reviews and primary research (both quantitative and qualitative)
- Identify evidence on the lived experience of TSWS in blogs and on posts on Instagram (Meta Platforms, Inc) and Reddit (Reddit, Inc)
- Identify gaps in evidence where further primary research is needed.

## Methods

### Design

An evidence gap map (EGM) is a systematic evidence synthesis product [30] that provides an overview of the available evidence on a particular topic, theme, or policy area. EGMs are used to highlight gaps in the evidence base, show where there is an abundance of evidence, and increase the discoverability and use of the evidence. EGM methodology seeks to show what evidence is available, not what the evidence says. As part of the “Big Picture” review family, Campbell et al [31] assert that “[n]o other review methodology has developed a systematic approach to identifying gaps in the evidence with this level of rigor and transparency.” This EGM aimed to identify the range, extent, and type of evidence about TSWS both in the research literature and on social media platforms. The scope of the EGM was defined by a years–research topics framework, with the rows as years and the columns as research topics. The framework was developed by examining the literature, drawing on the building blocks proposed by Howell et al [27] for high-quality research in TSW, and by consulting with our public collaborator (AS) who has lived experience of TSWS. Additional topics were added if identified within the included evidence. The social media evidence from Instagram and Reddit was restricted to 1 year, reflecting our decision to select a sample of the most recent posts using the popular hashtag #topicalsteroidwithdrawal on specific dates. This EGM has been conducted and reported according to Campbell guidelines for EGMs [30], and the EGM protocol was uploaded to Open Research Exeter, the web-based repository of the University of Exeter [32].

### Ethical Considerations

We carefully considered the ethical implications of this study and did not submit it for institutional review board approval because the study involved publicly available data and no analysis was undertaken.

### Patient and Public Involvement

Our public collaborator (AS), who has lived experience of TSWS, contributed throughout the process, from question development to manuscript preparation.

### Search Methods

Searches were conducted to identify published and unpublished literature via academic databases, websites, blogs, and social media (Instagram and Reddit).

#### Published and Unpublished Research

We searched MEDLINE and Embase (via Ovid), CINAHL (via EBSCOhost), and ProQuest Dissertations & Theses and Conference Proceedings Citation Index (CPCI-Science and CPCI-Social Science & Humanities via Web of Science) between August 17 and 26, 2022, using a combination of subject headings and free-text terms for steroids, topical application, and terms for withdrawal or addiction. The database searches were updated in November 2023. The full search strategies are available in Multimedia Appendix 1. We carried out forward and backward citation chasing of included studies using Epistemonikos (Epistemonikos Foundation; for reviews) and Scopus (Elsevier). We searched for the phrases “topical steroid withdrawal” and “topical steroid addiction” on Google using the approach recommended by Briscoe and Rogers [33] for additional relevant studies. All results were exported into EndNote (version 20; Clarivate) for screening.

#### Blogs

We searched for blogs using “topical steroid withdrawal” and “topical steroid addiction” in combination with the word “blogs” on Google. We also carried out a targeted search for “topical steroid withdrawal” on the blogging sites WordPress, Medium, and Blogspot. Blog searches were carried out in October 2022. There were no date or other limits applied to the blog searches. Searching for blogs as opposed to blog posts offered the possibility of understanding the experience of TSWS from a longitudinal perspective and following individuals through the withdrawal process [34].

#### Instagram and Reddit

We searched for the 100 most recent posts featuring #TopicalSteroidWithdrawal that included text on February 2, 2023. These were copied into an Excel (Microsoft Corp) spreadsheet for screening and coding, along with the date of the post and a URL link. Posts not relating to TSW, posts advertising products, posts that were not in English, or posts that only included links or hashtags were excluded. We searched for the 100 most recent and relevant posts under the subreddit Topical Steroid Withdrawal (r/TS_Withdrawal). The text, date, and URL were entered into an Excel spreadsheet for screening and coding. Searches on Reddit were carried out on February 7 and 8, 2023. Searching for the most recent and relevant posts on specific dates was chosen to simulate “real-world” Instagram and Reddit viewing behavior [35,36].

### Inclusion Criteria

Studies, blogs, and social media posts were included if they met the following criteria:

- Population: people experiencing TSWS (includes infants, children, and adolescents), either via complete “sudden” cessation of TCs or complete cessation of TCs via a “tapering” approach
- Exposure: TCs
- Outcomes: effects of withdrawal: physical, psychosocial, knowledge, and attitudes (among both health care professionals and people experiencing withdrawal) and information seeking and sharing by people experiencing withdrawal
- Study type/posts: any studies (quantitative or qualitative) that investigate TSW were included. Letters, case reports, commentaries, and opinion pieces were also included. Blogs or social media posts were included if they described any aspect of the experience of living with TSWS or caring for someone with TSWS.

### Exclusion Criteria

Studies were excluded if they reported side effects of corticosteroids that were not specific to withdrawal. Blogs and social media posts were excluded if they did not describe personal experience relating to TSWS. They were also excluded if they described and promoted treatments for TSWS that were not related to personal experience, for example, posts advertising products.

### Study Selection

Studies located via the database searches were exported into EndNote (version 9). Titles, abstracts, and full texts were screened independently by 2 reviewers (NO and MR). Disagreements were resolved by discussion. Posts from Instagram and Reddit were copied and pasted into an Excel spreadsheet and were screened independently by 2 reviewers. Details about blogs (URL, date coverage, number of posts, and usernames) were pasted into an Excel spreadsheet. Short summaries of the blogs were compiled and checked by 2 reviewers (NO and MR). Decisions about the inclusion of blogs were carried out during the data extraction and coding stage.

### Data Extraction and Mapping

Data extraction was conducted using EPPI-Reviewer [37]. The data extraction tool was informed by the research question and the structure of the map; it was piloted on a sample of included academic publications and social media posts and modified through team discussion. Data extraction was undertaken by 1 reviewer (NO) and checked by a second reviewer (MR), and any inconsistencies were resolved through discussion. Data were extracted on the type of evidence, location of evidence, type of review, study design, population (age), steroids used, duration of TC use, length of time “off” TCs, areas of body affected, reasons for stopping TCs, diagnosis, names of conditions, and research topics. Following best practice guidelines for conducting EGMs, we did not undertake quality appraisal [31].

EPPI-Reviewer [37] and EPPI-Mapper software [38] were used to generate the interactive EGM. The EGM uses a years–research topics framework (years as rows and research topics as columns), and within each cell of the grid, the evidence is presented as bubbles according to type, with the color and size of the bubble indicating the type and amount of evidence available within that cell of the grid, respectively. The filters of the map enable map users to change the type of evidence displayed based on, for example, authors, population age, and the location of evidence. Map users are also able to download lists of the studies from the map in total or by study type. Due to the nature of the included studies (ie, a study may comprise a review of the literature and a case report), a study may be represented in multiple places in the EGM.

## Results

### Results of the Search

The database searches identified 1914 records, of which, after duplicates were removed, 1248 titles and abstracts were screened. After excluding irrelevant records, 116 full texts were screened, with another 36 being screened following citation searching. Finally, 81 of the records were included in the map [5,9-18,23-27,29,39-101]).

**Figure 1:**
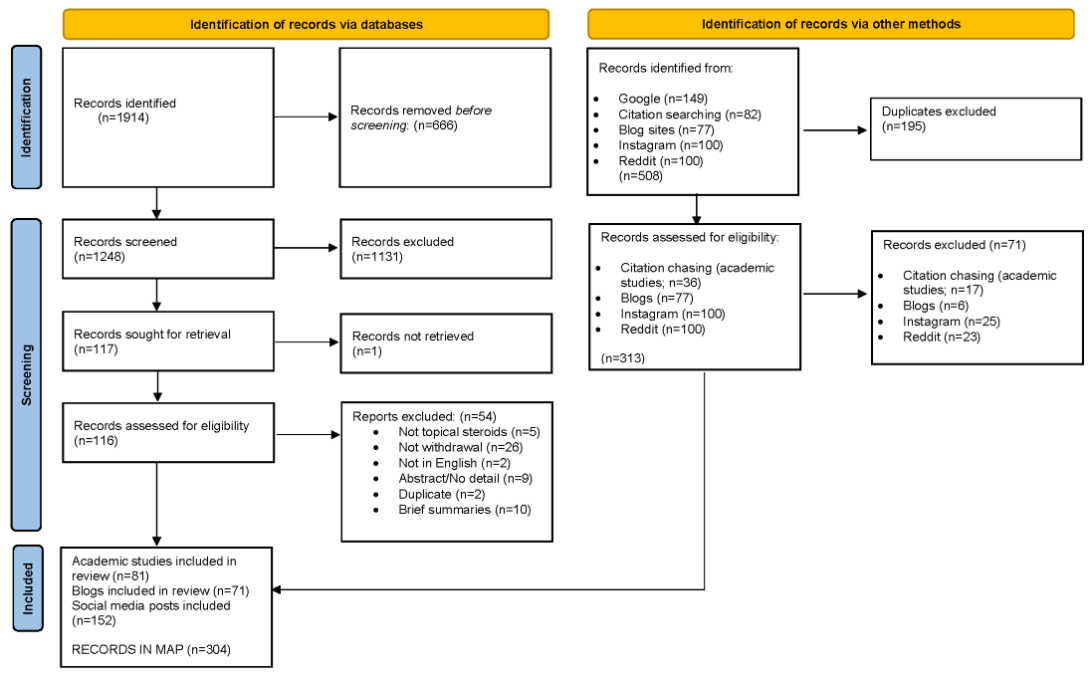
PRISMA (Preferred Reporting Items for Systematic Reviews and Meta-Analyses) diagram.

Of the 100 Instagram posts captured, 76 (76%) contained relevant information about the experience of TSW. Of the 100 Reddit posts captured, 77 (77%) contained relevant information about the experience of TSW. Of the 77 blogs identified through blog searching and internet searches, 71 (92%) met the inclusion criteria. The PRISMA (Preferred Reporting Items for Systematic Reviews and Meta-Analyses) diagram shows the flow of records throughout the screening process (Figure 1) [102].

### Results of the Map

The interactive EGM [103] comprises two distinct types of evidence: (1) research evidence published in academic journals and (2) evidence published by people (or their carers and family members) living with TSWS on social media. There were 81 publications by academics and clinicians and 223 social media posts (including Instagram posts, Reddit posts, and blogs) by people living with TSWS or by carers and family members.

**Figure 2:**
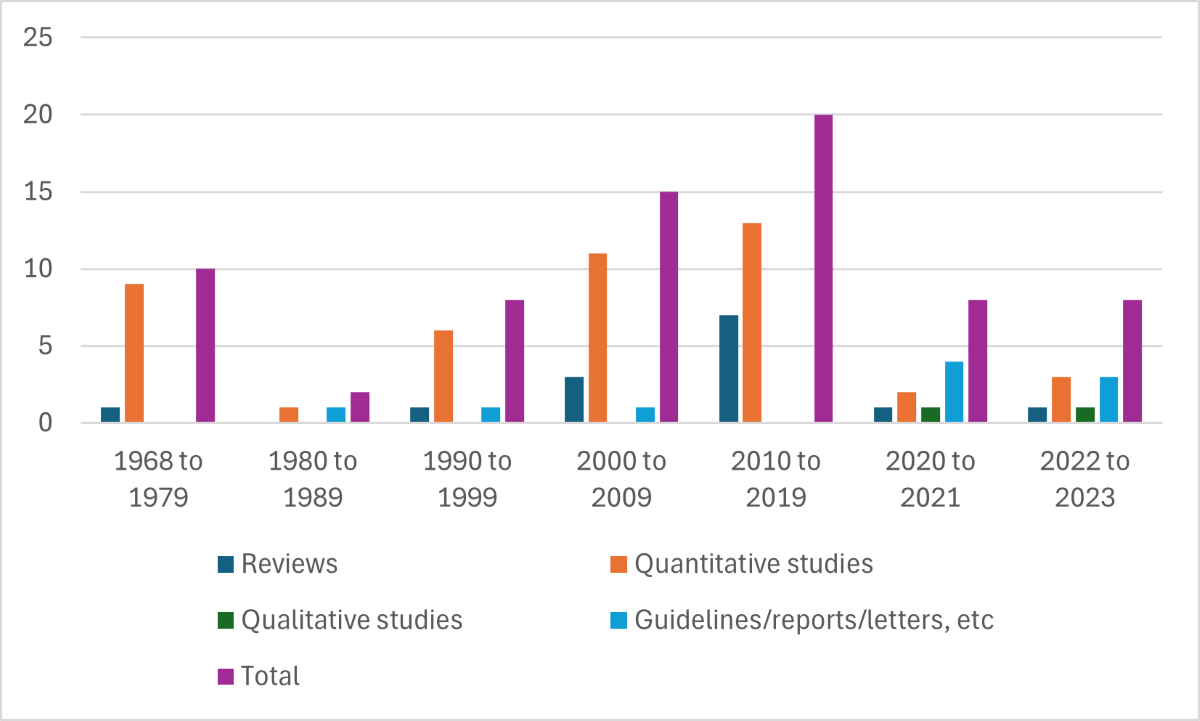
Number of academic publications and publication years.

The 81 publications by academics and clinicians were published across a period of 55 years from 1968 to 2023 (Figure 2), with 1 (1%) study published in 1969 [14] and increasing to 20 (24%) in 2022 to 2023. The research evidence published by academics and clinicians included reviews (16/81, 20%); quantitative studies (54/81, 66%); qualitative studies (2/81, 2%); and guidelines, reports, discussions, or editorials (10/81, 12%). Of the 16 reviews, only 2 (12%) were systematic reviews and 14 (88%) were nonsystematic reviews. The quantitative studies comprised mostly case reports or histories (34/54, 63%) and no randomized controlled trials. The research evidence was located in 14 countries: the United States (37/81, 46%), the United Kingdom (14/81, 17%), Australia (7/81, 9%), Japan (6/81, 7%), India (4/81, 5%), Taiwan (3/81, 4%), Singapore (2/81, 2%), Ireland (2/81, 2%), China (2/81, 2%), France (2/81, 2%), Canada (2/81, 2%), South Africa (1/81, 1%), Tunisia (1/81, 1%), and Germany (1/81, 1%).

Of 223 social media posts, there were 71 (31.8%) blogs and 152 (68.2%) Instagram and Reddit posts. The blogs were published between 2011 and 2022, and the Instagram and Reddit posts were published between 2022 and 2023, which reflects the decision to sample Instagram and Reddit posts on specific dates. The social media evidence was located in 11 countries: the United States (21/223, 9.4%), the United Kingdom (19/223, 8.5%), Australia (7/223, 3.1%), Canada (5/223, 2.2%), New Zealand (2/223, 0.9%), Singapore (2/223, 0.9%), Ireland (1/223, 0.4%), Taiwan (1/223, 0.4%), South Africa (1/223, 0.4%), Portugal (1/223, 0.4%), and Belgium (1/223, 0.4%). However, it was not always possible to know in which country the social media posts originated.

Most research evidence focused on adults (51/81, 63%), followed by children aged 4 to 12 years (18/81, 22%), infants aged 0 to 3 years (13/81, 16%), and young people aged 13 to 18 years (9/81, 11%). Likewise, the adult experience of TSWS was more highly represented in the social media evidence (125/223, 56.1%), with only 5.8% (13/223) of posts focusing on young people, children, and infants. Both the research evidence and the social media evidence did not always report the TCs used (40/81, 49% and 118/223, 52.9%, respectively), and Table 1 shows the TCs that were most used when specified.

It was not always possible to determine how long people had been using TCs, but 33 (41%) out of 81 publications reported people using TCs for up to 10 years and 13 (16%) reported people using TCs for >10 years. In the social media evidence, there were 25 (11.2%) out of 223 posts that indicated up to 10 years of use and 40 (17.9%) posts that indicated>10 years of use. The main areas of the body affected, as reported in the research evidence, were the face (40/81, 49%), legs (16/81, 20%), and arms (15/81, 19%). In the social media evidence, the main areas of the body affected were the face (50/223, 22.4%) and legs (25/223, 11.2%), but 22.4% (50/223) of the posts reported that the entire body was affected. The main reason for stopping TCs, according to the research evidence, was the physician’s advice to stop (10/81, 12%), followed by lack of durable benefit (6/81, 7%), lack of effectiveness (6/81, 7%), and the requirement to participate in a study (4/81, 5%). The main reason for stopping TCs, according to the social media evidence, was the awareness of TSWS as a condition (33/223, 14.8%); other reasons were lack of effectiveness (16/223, 7.2%), awareness of side effects (eg, skin thinning; 9/223, 4%), and lack of durable benefit (4/223, 1,8%). Diagnosis by a clinician or dermatologist was recorded mostly in the research evidence (26/81, 32%), while it was less common in the social media evidence (9/223, 4%). In contrast, self-diagnosis was most prevalent in social media (38/223, 17%), followed by diagnosis supported by social media (25/223, 11.2%). Patch testing was reported in both the research evidence (11/81, 14%) and social media evidence (2/223, 0.9%). There were also cases where a diagnosis of TSWS had not been confirmed in the social media evidence (9/223, 4%), where individuals reported that they were unsure whether they were experiencing TSWS or something else.

**Table 1:** Most used topical corticosteroids reported in research evidence and social media evidence (N=304).

Steroids used	Research evidence (n=81), n (%)	Social media evidence (n=223), n (%)
Betamethasone	28 (34.6)	22 (9.9)
Clobetasol	19 (23.5)	20 (9)
Hydrocortisone	16 (19.8)	36 (16.1)
Triamcinolone	15 (18.5)	13 (5.8)
Fluocinolone	13 (16)	0 (0)
Mometasone	13 (16)	18 (8.1)
Systemic steroids	13 (16)	16 (7.2)

### Overview

#### Risk Factors

The risk factors for TSWS featured in the research evidence and highlighted “prolonged use of moderate to high potency TCs” (34/81, 42%) and “prolonged and frequent use of TCs” (32/81, 40%) as the most recognized risk factors. History of atopy (15/81, 19%) and inflammatory skin conditions (12/81, 15%) were also reported. In contrast, risk factors did not feature strongly in the social media evidence, with only 4.9% (11/223) of posts referring to prolonged and frequent use of TCs.

#### Physical Symptoms (Skin and Nonskin)

A variety of skin symptoms of TSWS were reported in the academic publications: rash (including redness, papules, and erythema; 61/81, 75%), itch (39/81, 48%), burning or stinging (36/81, 44%), scaling (34/81, 42%), pustules (33/821 41%), telangiectasias (30/81, 37%), edema (30/81, 37%), and dryness and flaking (24/81, 30%). The social media evidence reported similar symptoms but with some variations: rash (9/223, 4%), itch (83/223, 37.2%), dryness or flaking (80/223, 35.9%), oozing or crustiness (69/223, 30.9%), burning or stinging (42/223, 18.8%), cuts (40/223, 17.9%), edema (38/223, 17%), skin pain (other than burning pain; 38/223, 17%), thickening of outer layer of skin (27/223, 12.1%), and smell (25/223, 11.2%).

The most common nonskin symptoms of TSWS reported in the research evidence were increased hair growth (11/81, 14%), sleep loss (9/81, 11%), temperature regulation (9/81, 11%), and lymphadenopathy (9/81, 11%). A wider range of symptoms were reported in the social media evidence with sleep loss (54/223, 24.2%), temperature regulation (38/223, 17%), fatigue and muscle weakness 29/223, 13%), pain (including nerve pain; 20/223, 9%), mobility issues (16/223, 7.2%), and lymphadenopathy (9/223, 4%).

#### Mental Health Symptoms

In the research evidence, 12% (10/82) of the academic publications reported depression, anxiety, and stress; 7% (6/81) reported suicidal thoughts; and 6% (5/81) reported emotional fluctuations. Interestingly, the social media evidence captured 2 additional aspects of mental health, including self-image (both negative and positive; 45/223, 20.2%) and resilience (45/223, 20.2%). These were followed by depression, anxiety, and stress (42/223, 18.8%); emotional fluctuations (25/223, 11.2%); and suicidal thoughts 4/223, 1.8%).

#### Relationships

How living with TSWS impacted adults’ and children’s relationships with others was not addressed in the research evidence, apart from 1 qualitative study that acknowledged its impact on relationships within the home and with physicians and dermatologists. In contrast, this featured strongly within the social media evidence, including relationships at home (45/223, 20.2%), at work, and in education (7/223, 3.1%), as well as relationships with physicians and dermatologists (45/223, 20.2%), with other therapists (9/223, 4%), and with other people with TSWS often via social media (31/223, 13.9%).

#### Beliefs and Attitudes

The beliefs and attitudes toward TCs, TSWS, and information on TCs and TSWS featured most strongly in the social media evidence. The belief in the importance of perseverance for coping with TSWS was emphasized (49/223, 22%), followed by the need for “alternative” sources of information on TSWS (31/223, 13.9%) and the belief that the information on TCs from physicians was inadequate (20/223, 9%). Mistrust of medical professionals featured (20/223, 9%), along with the perception that physicians do not believe that TSWS exists (16/223, 7.2%) and fears of steroids and medications (11/223, 4.9%). The research evidence was sparse but fear of steroids and medications (6/81, 7%), alternative sources of information on TSWS (5/81, 6%), and belief that TSWS does not exist (5/81, 6%) were reported.

#### Activities of Everyday Living

Living with TSWS affected people’s everyday lives, and not surprisingly, this was captured by the social media evidence across all the domains: social life (31/223, 13.9%), work (31/223, 13.9%), self-care (25/223, 11.2%), holidays and leisure (13/223, 5.8%), and college or school (11/223, 4.9%). In contrast, the research evidence gave little attention to how TSWS impacted work (3/81, 4%), college or school (3/81, 4%), and social life (2/81, 2%).

#### Treatments

Complete cessation of TCs was the favored strategy in both the research evidence (48/81, 59%) and the social media evidence (51/223, 22.9%). Tapering of TCs featured more in the research evidence (22/81, 27%) than in the social media evidence (11/223, 4.9%). Using systemic steroids as a treatment was reported in the research evidence (11/81, 14%) but less in the social media evidence (4/223, 1.8%), and restarting TCs was reported in the social media (8/223, 3.6%) but to a lesser extent in the research evidence (5/81, 6%). Some of the pharmacological treatments mentioned in the research evidence were niche (mentioned only once), such as Montelukast, adrenocorticotropic hormone, platelet-rich plasma, intravenous immunoglobulin, and Nicardipine, and were not mentioned in the social media evidence at all. Not surprisingly, the research evidence tended to report greater use of pharmacological treatments, while the social media evidence highlighted the treatments most used by people living with TSWS, as presented in Table 2.

**Table 2:** Treatments for topical steroid withdrawal syndrome in research evidence and social media evidence (N=304).

Treatments used	Research evidence (n=81), n (%)	Social media evidence (n=223), n (%)
Antihistamines	23 (28)	23 (10.3)
Antibiotics	33 (40.7)	20 (9)
Cyclosporine	8 (9.9)	9 (4)
Dupilumab	8 (9.9)	11 (4.9)
Methotrexate	4 (4.9)	4 (1.8)
Emollients and/or moisturizers	25 (30.9)	58 (26)
Alternative remedies	8 (9.9)	51 (22.9)
Bathing interventions	5 (6.2)	47 (21.1)
Diet changes	10 (12.3)	29 (13)
Clothing interventions	1 (1.2)	20 (9)
Hot and/or cold treatments	15 (18.5)	20 (9)
Psychological therapy and/or support	18 (22)	1 (0.5)
Online support	4 (4.9)	36 (16.1)

#### Treatment Costs and Outcome Measures

Treatment costs were only mentioned in a small proportion of the social media evidence (16/223, 7.2%) and research evidence (2/81, 2%). There was little on outcome measures, with 5% using the Dermatology Life Quality Index and 1% using self-assessed questionnaires. The social media evidence showed that people were recording their feelings of progress on the “TSW journey” to recovery (22/223, 9.9%), and a smaller proportion (9/223, 4%) referred to “being healed” as reaching the end of the TSW journey.

## Discussion

### Principal Findings

This EGM presented the developing body of research evidence on TSWS, enabling a timely insight into the topics of interest to the medical and dermatological community. The research evidence was distributed across a number of topics, but there were many where evidence was clearly lacking, such as diagnosis, prevention, epidemiology, relationships, activities of everyday living, beliefs and attitudes, treatment costs, and outcome measures. The EGM also presented a sample of the social media evidence on TSWS, which concentrated on the physical symptoms (skin and nonskin), mental health symptoms, relationships, activities of everyday living, beliefs and attitudes, and treatments. This, by implication, highlights additional topics that need greater attention from academics and clinicians and confirms the need for research on the patient’s lived experience of TSWS.

The lack of research evidence published across the topics of diagnosis, prevention, and epidemiology is not surprising, given that the legitimacy of the condition is still questioned by many in the medical and dermatological communities [15,29]. The need for consensus on diagnostic criteria has been recognized as a “priority” by a few authors [27,39], and arguably, without these, it is not possible to understand the incidence, prevalence, and distribution of TSWS. In 2014, Fukaya et al [13] stated that there were “no statistics regarding the prevalence of TSA,” and this remains the case. The challenge of diagnosing TSWS was reiterated [12,16,17,20,40,41], and the main differential diagnoses were atopic dermatitis itself, allergic contact dermatitis, and infection. Sheary [12] observed that many of the symptoms of TSWS may also be seen in severe atopic dermatitis but argued that the process of differential diagnosis relies on a thorough understanding of patient history and physical examination. Unsurprisingly, most cases of TSWS are self-diagnosed rather than by a clinician or dermatologist [17], which was confirmed by the social media evidence. Sheary [12] proposed a set of diagnostic criteria as a starting point for discussion and future research; it comprised (1) essential criteria, (2) key diagnostic criteria, and (3) additional supporting features that may be present. The essential criteria are history of long-term regular use of TCs, itch, and erythema. Lio and Chandan’s [17] key diagnostic criteria (equivalent to the “essential” criteria proposed by Sheary [12]) were burning, confluent erythema, and a history of frequent and prolonged TC use. These proposals, along with the work on diagnostic criteria proposed by Guo et al [104] using a modified Delphi approach, could provide a way forward for the medical and dermatological communities to engage with the issue of agreeing on recognized diagnostic criteria.

Given that addiction precedes withdrawal, prevention of addiction to TCs is of paramount importance. According to Sheary [18], it is “not widely accepted” within the medical community that there may be withdrawal symptoms associated with TC cessation, and she argues that this may be because TCs have been traditionally recommended for “intermittent flares of symptoms” rather than long-term use. However, the EGM indicates that patients can use TCs for years. For example, in a recent survey, people with eczema reported using TCs for >15 years [42], and in another study, the length of use of TCs for some people was reported to be ≥40 years [12]. Sheary [18] observed that TC “overuse” has never been defined, and arguably, neither has “prolonged use.” That research has not investigated prolonged use of TCs has been shown by a recent “umbrella review” on the safety of TCs in adults and children with eczema [106], which found limited high-quality evidence for long-term use of TCs. The data on prolonged use were limited as many randomized controlled trials were of short duration and did not include follow-up beyond 2 to 4 weeks of treatment. This echoes Eichenfield et al’s [2] observations in 2014 that “...most studies fail to follow up patients for potential complications,” and despite their recommendation that treatment sites should be monitored regularly for adverse effects, just over half of the international guidelines in the review by LePoidevin et al [1] recommended screening for cutaneous side effects. Axon et al [105] recommended that “...longer-term prospect observational studies are better placed to exploit longer-term safety of TCs and should be designed with years rather than months of follow-up to add useful information to the field.” Understanding the impact and safety of prolonged use of TCs has not been prioritized, as argued by Peacock [106], who contends that large-scale research funding has, for decades, primarily focused on product development for pharmaceutical companies.

From the existing research evidence in this EGM, prolonged and frequent use of TCs and prolonged use of moderate or high potency TCs were identified as risk factors for TSWS. Yet, this has to be juxtaposed with the dominant focus of the published medical literature on the safety of TCs, reporting on patient “misuse/abuse,” “inappropriate use,” or “underuse” [107]; and how to encourage “correct use” [108]. The “reluctance to use topical steroids as recommended” [109] has been dismissed by many dermatologists as “steroid phobia,” as has TSWS [15]. Arguably, this narrative of blame filters through to general practitioners (GPs) who can “struggle” [106] to support people experiencing TSWS and need guidance from dermatologists on the safe use of TCs. As already noted, there is considerable diversity across the international management guidelines for atopic dermatitis on the use of TCs. For example, the guidance on optimal dosage is variable [1], not least because the evidence base lacks “...studies that examine a range of TCS doses in large numbers of patients” [2]. In the UK context, the MHRA provided advice for health care professionals in 2021 [20] on prescribing TCs in terms of potency, quantity to be applied, and area, frequency, and duration of application. The advice included reporting suspected adverse drug reactions to the Yellow Card Scheme (a UK scheme for monitoring suspected side effects to medicines and medical devices) after cessation of TCs. A recent update to the advice [110] details that TCs will be labeled with information on their potency to help with counseling patients. Given the key role GPs have in prescribing TCs, there is a case to be made for research on how to implement training and guidance for GPs and other health care professionals as an important part of building the evidence base on the safe use of TCs.

The research evidence devoted considerable attention to the physical “skin” symptoms of TSWS, which concurred, in the main, with the social media evidence. However, the social media evidence expanded on “other” physical symptoms, mental health symptoms of TSWS, treatments used, and how it impacted activities of everyday living and relationships. This gap in the research evidence can be explained by the lack of qualitative research with only 2 qualitative studies on TSWS published in the last 50 years. The research evidence also addressed treatments to manage TSWS, which tended to be pharmacological, with some of these treatments also mentioned in social media. The social media evidence revealed that people with TSWS were using a range of nonpharmacological treatments, which included emollients and moisturizers and bathing interventions, and then “alternative remedies,” which could encompass acupuncture, hypnotherapy, and homeopathy, for example. Interestingly, the research evidence on pharmacological treatments such as Dupilumab reported treatment improvements after relatively short periods, such as 8, 13, and 31 weeks [43]. “Successful” treatments were less “linear” in the social media evidence, particularly in the blogs, where people with TSWS often reported a “roller coaster” experience of recovery for many months and even years, which could be punctuated with debilitating flares of symptoms such as itch, flaking, and oozing. With only 1 prospective cohort study in the EGM [23], there is clearly a need for more longitudinal research on the patient’s “TSWS journey” to healing.

Psychological therapy and support was one of the treatments that clinicians recommended for people with TSWS, but this hardly featured in the social media evidence. Online support was important for people with TSWS, which is possibly an artifact of social media being a form of online support and a reflection of the quality of the physician-patient relationship. Those who felt that their experiences and concerns were disregarded by their physicians and dermatologists often turned to social media for support and information [26].

Arguably, regardless of whether dermatologists and clinicians accept TSWS as a distinct clinical entity, there are important reasons why they need to engage with patients’ stories of living with TSWS from social media. To dismiss people living with TSWS as “misusers” and “misinformed” means that they may be left alone to deal with the symptoms of TSWS and consequently may “seek inappropriate alternative therapies”^’^ [39] that could be detrimental to their care and impact their recovery from TSWS [9]. Furthermore, dismissal by medical professionals has been found to introduce another element of distress to the lives of people with TSWS: in short, people living with TSWS “need to be heard and acknowledged by the medical community” [44].

### Strengths and Limitations

This study represents the first EGM in TSWS and responds to calls for a better understanding of TSWS. It aims to be an important resource to guide both researchers and clinicians in the prioritization of research topics for further research. Support from our public collaborator meant that this study was grounded in “real” lived experience, which is of great importance for ensuring that research is “meaningful to patients” [27]. Additional collaborative input from a researcher or clinician with direct dermatological expertise would also have been beneficial. Mapping both research evidence and social media evidence is a methodological innovation in the production of EGMs. The inclusion of social media evidence was in recognition of the increased presence of #topicalsteroidwithdrawal on social media [24] and how social media content could contribute to understanding the patient perspective of TSWS. Social media is increasingly used by qualitative health researchers, and there has been some interest in social media within the dermatological community [111]. In the EGM, there is only 1 example of researchers reviewing social media blogs to understand children’s experiences of TSWS [45]. Due to the amount of social media content available on TSWS, we decided that a sample of social media evidence was the only feasible approach for this EGM, and therefore, it provides a single snapshot of the social media evidence. Furthermore, bloggers and people using Instagram and Reddit are a self-selecting sample who are clearly proficient with technology, so it is difficult to know how reflective they are of those living with TSWS. This does not devalue their accounts but means that there are likely to be many people’s experiences of TSWS that are not shared on social media. There were also some cases, bloggers in particular, who presented with a self-diagnosis of TSWS but were unsure whether their symptoms were indicative of TSWS, and it was challenging to judge the veracity of their accounts. The ephemeral nature of social media data should also be noted, and some of our selected Instagram and Reddit posts were removed after inclusion in the EGM. As an EGM dealing with a range of academic publications and a sample of social media evidence, we did not conduct a critical appraisal [31]. The low quality of the existing research evidence has been noted [9,16,25], and there is undoubtedly an urgent need for high-quality qualitative and quantitative research. However, this EGM offers an overview of the TSWS research landscape and an insight into what people living with TSWS are discussing on social media.

### Conclusions

TSA, as an adverse effect of TCs, was first identified in 1969; and yet, >50 years later, TSWS remains controversial and contested in the dermatological and medical communities. This EGM shows that TSWS has attracted increased research attention over the years, but high-quality research is lacking. The evidence gaps highlight priorities for future primary research, which are longer-term prospective observational studies to assess the safety of prolonged use of TCs and help prevent addiction, qualitative research to understand the lived experience of TSWS, and longitudinal research on the patient’s “TSWS journey” to healing. However, it is crucial that future research in TSWS is underpinned by work that determines agreed diagnostic criteria for TSWS and by collaboration between researchers and clinicians with the TSWS patient community.

## Appendix

Multimedia Appendix 1 Search strategy.

## Abbreviations

EGM: evidence gap map
GP: general practitioner
MHRA: Medicines and Healthcare products Regulatory Agency
PRISMA: Preferred Reporting Items for Systematic Reviews and Meta-Analyses
TC: topical corticosteroid
TSA: topical steroid addiction
TSW: topical steroid withdrawal
TSWS: topical steroid withdrawal syndrome
