# Supplementary material for: Reviewing the Evidence Base for Topical Steroid Withdrawal Syndrome in the Research Literature and Social Media Platforms: An Evidence Gap Map
Source: J Med Internet Res. 2024 Dec 6;26:e57687. doi: 10.2196/57687 (PMC11662184; doi:10.2196/57687)
Supplement: Multimedia Appendix 1 [file jmir_v26i1e57687_app1.docx]

**Multimedia Appendix 1**

**Database search strategies**

EMBASE via Ovid (same terms used for MEDLINE)

Embase <1974 to 2022 August 25>

1 topical.ti,ab. 149428

2 cream*.ti,ab. 31826

3 ointment*.ti,ab. 17307

4 1 or 2 or 3 180212

5 steroid*.ti,ab. 357164

6 corticosteroid*.ti,ab. 172949

7 Alclometasone.ti,ab. 83

8 Amcinonide.ti,ab. 99

9 (Beclometasone or beclomethasone).ti,ab. 4519

10 Betamethasone.ti,ab. 7363

11 clobetasone.ti,ab. 210

12 Clobetasol.ti,ab. 2099

13 Desonide.ti,ab. 173

14 Desoximetasone.ti,ab. 114

15 Diflucortolone.ti,ab. 163

16 Diflorasone.ti,ab. 77

17 Fluocinolone.ti,ab. 1086

18 Fluocinonide.ti,ab. 249

19 Flurandrenolide.ti,ab. 30

20 Fluticasone.ti,ab. 7601

21 Halcinonide.ti,ab. 117

22 Halobetasol.ti,ab. 138

23 Hydrocortisone.ti,ab. 20371

24 Methylprednisolone.ti,ab. 30251

25 Mometasone.ti,ab. 1926

26 Triamcinolone.ti,ab. 10694

27 aclovate.ti,ab. 3

28 cyclocort.ti,ab. 2

29 (Audavate or Betnovate or Diprosone or Diprolene or Diprovate or Fucicort or Daivobet).ti,ab. 212

30 (Clobaderm or Dermovate).ti,ab. 148

31 Eumovate.ti,ab. 22

32 (Tridesilon or DesOwen).ti,ab. 2

33 Topicort.ti,ab. 6

34 (Nerisone or Neriderm).ti,ab. 11

35 Psorcon.ti,ab. 3

36 (Synalar or Synemol or Fluonid or Dermasmooth or Derma-Smoothe).ti,ab. 41

37 Lidex.ti,ab. 11

38 (Haelan or Cordran).ti,ab. 16

39 Cutivate.ti,ab. 19

40 Halog.ti,ab. 5

41 (Ultravate or Halox).ti,ab. 8

42 (Mildison or Dermacort or Westcort or Locoid or Hytone or Dioderm or HC45 or Zenoxone or Lanacort or Hydrocort or Alphosyl or Aquacort).ti,ab. 97

43 Advantan.ti,ab. 36

44 Elocon.ti,ab. 28

45 (Aristocort or Viaderm or Kenocomb).ti,ab. 11

46 or/5-45 551938

47 (addicted or addiction).ti,ab. 77452

48 (withdrawal or withdrew or withdrawn).ti,ab. 183459

49 (abuse or abusing or misuse or misusing).ti,ab. 201360

50 47 or 48 or 49 434784

51 4 and 46 and 50 975

52 red skin syndrome.ti,ab. 5

53 red face syndrome.ti,ab. 8

54 steroid rosacea.ti,ab. 21

55 steroid dermatitis.ti,ab. 9

56 steroid dependent dermatitis.ti,ab. 2

57 corticosteroid dependent dermatitis.ti,ab. 7

58 (corticosteroid induced adj3 dermatitis).ti,ab. 10

59 corticosteroid addictive dermatitis.ti,ab. 4

60 (corticosteroid induced adj2 rosacea).ti,ab. 9

61 (steroid induced adj2 rosacea).ti,ab. 40

62 (steroid induced adj2 dermatitis).ti,ab. 17

63 52 or 53 or 54 or 55 or 56 or 57 or 58 or 59 or 60 or 61 or 62 118

64 51 or 63 1074

CINAHL

S71 S58 OR S70

S70 S59 OR S60 OR S61 OR S62 OR S63 OR S64 OR S65 OR S66 OR S67 OR S68 OR S69

S69 TI steroid induced N2 dermatitis OR AB steroid induced N2 dermatitis

S68 TI steroid induced N2 rosacea OR AB steroid induced N2 rosacea

S67 TI corticosteroid induced N2 rosacea OR AB corticosteroid induced N2 rosacea

S66 TI corticosteroid addictive dermatitis OR AB corticosteroid addictive dermatitis

S65 TI (corticosteroid induced N3 dermatitis) OR AB (corticosteroid induced N3 dermatitis)

S64 TI "corticosteroid dependent dermatitis" OR AB "corticosteroid dependent dermatitis"

S63 TI "steroid dependent dermatitis" OR AB "steroid dependent dermatitis"

S62 TI "steroid dermatitis" OR AB "steroid dermatitis"

S61 TI "steroid rosacea" OR AB "steroid rosacea"

S60 TI "red face syndrome" OR AB "red face syndrome"

S59 TI "red skin syndrome" OR AB "red skin syndrome"

S58 S4 AND S53 AND S57

S57 S54 OR S55 OR S56

S56 ((TI abuse OR AB abuse) OR (TI abusing OR AB abusing) OR (TI misuse OR AB misuse) OR (TI misusing OR AB misusing))

S55 ((TI withdrawal OR AB withdrawal) OR (TI withdrew OR AB withdrew) OR (TI withdrawn OR AB withdrawn))

S54 ((TI addicted OR AB addicted) OR (TI addiction OR AB addiction))

S53 S5 OR S6 OR S7 OR S8 OR S9 OR S10 OR S11 OR S12 OR S13 OR S14 OR S15 OR S16 OR S17 OR S18 OR S19 OR S20 OR S21 OR S22 OR S23 OR S24 OR S25 OR S26 OR S27 OR S28 OR S29 OR S30 OR S31 OR S32 OR S33 OR S34 OR S35 OR S36 OR S37 OR S38 OR S39 OR S40 OR S41 OR S42 OR S43 OR S44 OR S45 or S46 or S47 or S48 or S49 or S50 or S51 or S52

S52 ((TI Aristocort OR AB Aristocort) OR (TI Viaderm OR AB Viaderm) OR (TI Kenocomb OR AB Kenocomb))

S51 (TI Elocon OR AB Elocon)

S50 (TI Advantan OR AB Advantan)

S49 ((TI Mildison OR AB Mildison) OR (TI Dermacort OR AB Dermacort) OR (TI Westcort OR AB Westcort) OR (TI Locoid OR AB Locoid) OR (TI Hytone OR AB Hytone) OR (TI Dioderm OR AB Dioderm) OR (TI HC45 OR AB HC45) OR (TI Zenoxone OR AB Zenoxone) OR (TI Lanacort OR AB Lanacort) OR (TI Hydrocort OR AB Hydrocort) OR (TI Alphosyl OR AB Alphosyl) OR (TI Aquacort OR AB Aquacort))

S48 ((TI Ultravate OR AB Ultravate) OR (TI Halox OR AB Halox))

S47 (TI Halog OR AB Halog)

S46 (TI Halog OR AB Halog)

S45 (TI Cutivate OR AB Cutivate)

S44 (TI Cutivate OR AB Cutivate)

S43 ((TI Haelan OR AB Haelan) OR (TI Cordran OR AB Cordran))

S42 (TI Lidex OR AB Lidex)

S41 ((TI Synalar OR AB Synalar) OR (TI Synemol OR AB Synemol) OR (TI Fluonid OR AB Fluonid) OR (TI Dermasmooth OR AB Dermasmooth) OR (TI Derma-Smoothe OR AB Derma-Smoothe))

S40 (TI Psorcon OR AB Psorcon)

S39 (TI Psorcon OR AB Psorcon)

S38 ((TI Nerisone OR AB Nerisone) OR (TI Neriderm OR AB Neriderm))

S37 ((TI Nerisone OR AB Nerisone) OR (TI Neriderm OR AB Neriderm))

S36 (TI Topicort OR AB Topicort)

S35 (TI Topicort OR AB Topicort)

S34 ((TI Tridesilon OR AB Tridesilon) OR (TI DesOwen OR AB DesOwen))

S33 ((TI Tridesilon OR AB Tridesilon) OR (TI DesOwen OR AB DesOwen))

S32 (TI Eumovate OR AB Eumovate)

S31 ((TI Clobaderm OR AB Clobaderm) OR (TI Dermovate OR AB Dermovate))

S30 ((TI Audavate OR AB Audavate) OR (TI Betnovate OR AB Betnovate) OR (TI Diprosone OR AB Diprosone) OR (TI Diprolene OR AB Diprolene) OR (TI Diprovate OR AB Diprovate) OR (TI Fucicort OR AB Fucicort) OR (TI Daivobet OR AB Daivobet))

S29 (TI cyclocort OR AB cyclocort)

S28 (TI cyclocort OR AB cyclocort)

S27 (TI aclovate OR AB aclovate)

S26 (TI Triamcinolone OR AB Triamcinolone)

S25 (TI Mometasone OR AB Mometasone)

S24 (TI Methylprednisolone OR AB Methylprednisolone)

S23 (TI Hydrocortisone OR AB Hydrocortisone)

S22 (TI Halobetasol OR AB Halobetasol)

S21 (TI Halcinonide OR AB Halcinonide)

S20 (TI Fluticasone OR AB Fluticasone)

S19 (TI Flurandrenolide OR AB Flurandrenolide)

S18 (TI Fluocinonide OR AB Fluocinonide)

S17 (TI Fluocinolone OR AB Fluocinolone)

S16 (TI Diflorasone OR AB Diflorasone)

S15 (TI Diflucortolone OR AB Diflucortolone)

S14 (TI Desoximetasone OR AB Desoximetasone)

S13 (TI Desonide OR AB Desonide)

S12 (TI Clobetasol OR AB Clobetasol)

S11 (TI clobetasone OR AB clobetasone)

S10 (TI Betamethasone OR AB Betamethasone)

S9 ((TI Beclometasone OR AB Beclometasone) OR (TI beclomethasone OR AB beclomethasone))

S8 (TI Amcinonide OR AB Amcinonide)

S7 (TI Alclometasone OR AB Alclometasone)

S6 (TI corticosteroid* OR AB corticosteroid*)

S5 (TI steroid* OR AB steroid*)

S4 S1 OR S2 OR S3

S3 (TI ointment* OR AB ointment*)

S2 (TI cream* OR AB cream*)

S1 (TI topical OR AB topical)

ProQuest Theses & Dissertations

((TI,AB(topical)) OR (TI,AB(cream*)) OR (TI,AB(ointment*)) ) AND ((TI,AB(steroid*)) OR (TI,AB(corticosteroid*)) OR (TI,AB(Alclometasone)) OR (TI,AB(Amcinonide)) OR ((TI,AB(Beclometasone) OR TI,AB(beclomethasone)) ) OR (TI,AB(Betamethasone)) OR (TI,AB(clobetasone)) OR (TI,AB(Clobetasol)) OR (TI,AB(Desonide)) OR (TI,AB(Desoximetasone)) OR (TI,AB(Diflucortolone)) OR (TI,AB(Diflorasone)) OR (TI,AB(Fluocinolone)) OR (TI,AB(Fluocinonide)) OR (TI,AB(Flurandrenolide)) OR (TI,AB(Fluticasone)) OR (TI,AB(Halcinonide)) OR (TI,AB(Halobetasol)) OR (TI,AB(Hydrocortisone)) OR (TI,AB(Methylprednisolone)) OR (TI,AB(Mometasone)) OR (TI,AB(Triamcinolone)) OR (TI,AB(aclovate)) OR (TI,AB(cyclocort)) OR ((TI,AB(Audavate) OR TI,AB(Betnovate) OR TI,AB(Diprosone) OR TI,AB(Diprolene) OR TI,AB(Diprovate) OR TI,AB(Fucicort) OR TI,AB(Daivobet)) ) OR ((TI,AB(Clobaderm) OR TI,AB(Dermovate)) ) OR (TI,AB(Eumovate)) OR ((TI,AB(Tridesilon) OR TI,AB(DesOwen)) ) OR (TI,AB(Topicort)) OR ((TI,AB(Nerisone) OR TI,AB(Neriderm)) ) OR (TI,AB(Psorcon)) OR ((TI,AB(Synalar) OR TI,AB(Synemol) OR TI,AB(Fluonid) OR TI,AB(Dermasmooth) OR TI,AB(Derma-Smoothe)) ) OR (TI,AB(Lidex)) OR ((TI,AB(Haelan) OR TI,AB(Cordran)) ) OR (TI,AB(Cutivate)) OR (TI,AB(Halog)) OR ((TI,AB(Ultravate) OR TI,AB(Halox)) ) OR ((TI,AB(Mildison) OR TI,AB(Dermacort) OR TI,AB(Westcort) OR TI,AB(Locoid) OR TI,AB(Hytone) OR TI,AB(Dioderm) OR TI,AB(HC45) OR TI,AB(Zenoxone) OR TI,AB(Lanacort) OR TI,AB(Hydrocort) OR TI,AB(Alphosyl) OR TI,AB(Aquacort)) ) OR (TI,AB(Advantan)) OR (TI,AB(Elocon)) OR ((TI,AB(Aristocort) OR TI,AB(Viaderm) OR TI,AB(Kenocomb)) ) ) AND (((TI,AB(addicted) OR TI,AB(addiction)) ) OR ((TI,AB(withdrawal) OR TI,AB(withdrew) OR TI,AB(withdrawn)) ) OR ((TI,AB(abuse) OR TI,AB(abusing) OR TI,AB(misuse) OR TI,AB(misusing)) ) )
